# Targeted Therapies in Low-Grade Serous Ovarian Cancers

**DOI:** 10.1007/s11864-024-01205-4

**Published:** 2024-06-13

**Authors:** Anna Gonzalez, Christa I. Nagel, Paulina J. Haight

**Affiliations:** Division of Gynecologic Oncology, Department of Obstetrics and Gynecology, The Ohio State University Wexner Medical Center, James Hospital and Solove Research Institute, M-210 Starling Loving Hall, 320 W. 10th Avenue, Columbus, OH 43210 USA

**Keywords:** Low-grade serous ovarian cancer

## Abstract

Low grade serous carcinoma of the ovary has been delineated as a separate entity from its counterpart high grade serous carcinoma of the ovary. Molecular profiling has helped to further characterize this disease process and has led to new and exciting treatment options. Surgery has always been a cornerstone of management both in primary and recurrent disease settings. Chemotherapy has been a long-standing backbone of adjuvant treatment, but its efficacy continues to be questioned. Hormonal therapy for upfront and recurrent disease is an effective treatment option with a high response rate and minimal side effects. Newer therapies including MEK, CDK 4/6, and PI3KCA inhibitors have emerged as exciting options for recurrent disease. Ongoing clinical trials will hopefully lead to additional therapeutic opportunities based on novel biomarkers in this disease.

## Introduction

Low grade serous ovarian carcinoma (LGSOC) is uncommon, comprising only about 2% of epithelial ovarian cancers [1, 2]. While previously grouped with its high-grade serous counterpart, more contemporary data supports the distinction of LGSOC as a unique clinical entity. Molecular profiling of LGSOC has become instrumental for our understanding of disease biology and the development targeted therapeutics for a tumor type classically thought to be chemoresistant [3••].

The majority (60%) of LGSOC cases are associated with a serous borderline neoplasm, which is likely a precursor lesion. Still, a significant proportion of cases arise de novo and may be ovarian or primary peritoneal, the latter of which is associated with improved prognosis [4•]. When compared to patients with high grade serous ovarian carcinoma (HGSOC), those with LGSOC tend to be younger, have higher BMI, have lower CA-125 levels, and are less likely to have ascites [5]. Patients with LGSOC may have more indolent onset of symptoms, but still most are diagnosed at advanced stages (FIGO II-IV). While professional societies recommend germline genetic testing for all patients with epithelial ovarian cancer, the likelihood of LGSOC being associated with a genetic predisposition is low, < 5% [6–8]. Somatic testing of LGSOC also reveals low rates of genetic mutation associated with homologous recombination deficiency [9, 10]. Somatic MAPK pathway alteration has been associated with improved survival outcomes, in addition to older age, lower BMI, and lack of tobacco use [4•, 8, 11, 12].

Surgery is the mainstay of treatment for LGSOC. Primary surgical management is the same as that for ovarian cancer in general, where those patients with apparent early-stage disease undergo staging and those with advanced-stage disease undergo debulking [13]. Completeness of cytoreduction is an important prognostic factor. In an ancillary analysis of GOG 182, only residual disease at the time of surgery was significantly associated with survival on multivariate analysis (median PFS 33.2 vs 14.1 months for those with microscopic vs macroscopic residual disease, respectively), highlighting the importance of optimal tumor debulking if possible [5]. Adjuvant chemotherapy is often given following surgical resection despite relatively low response rates of LGSOC to standard cytotoxic chemotherapy [14]. Unlike its high-grade serous counterpart where response rates to platinum-based chemotherapy reach 90%, LGSOC has been associated with low overall response rates of 4–20% [15–17].

Most patients (> 70%) diagnosed with advanced-stage LGSOC will recur. Given the relative chemoresistance of LGSOC, treatment in the recurrent setting often focuses on a combination of secondary cytoreduction (when feasible), hormonal therapy, targeted therapy, and/or clinical trial [3••, 6]. The pathologic and molecular features of the tumor often drive these treatment decisions. Most are hormone-receptor positive (90% estrogen receptor [ER], 50% progesterone receptor [PR]), making progestins or aromatase inhibitors particularly enticing in this setting [18, 19]. Additionally, a significant proportion of LGSOC tumors have KRAS (40%) or BRAF (5%) molecular alterations, highlighting the dominant role of the MAPK pathway in tumor biology and unveiling an important therapeutic target [8, 20, 21]. Clinical trials currently enrolling patients with recurrent LGSOC focus on these and other molecular alterations, highlighting the importance of precision medicine in the LGSOC treatment paradigm. Based on results of these trials, targeted therapy may become more prominent in the frontline setting.

## Treatment

### Surgery

Surgery is the preferred primary treatment strategy for LGSOC [6, 7]. Surgical staging for patients with early-stage disease includes hysterectomy, bilateral salpingo-oophorectomy, omentectomy, pelvic and para-aortic lymphadenectomy, peritoneal washings and biopsies [22]. Fertility-sparing surgery may be an option for patients with stage IA-IC1 LGSOC [3••, 23]. For those with advanced-stage disease, complete gross resection is associated with improved survival [5, 11, 16]. In an ancillary analysis of GOG 182, Fader et al. reported that residual disease was the only factor independently associated with PFS (HR 2.28) and OS (HR 2.12) [5]. Grabowski et al. reported on an exploratory analysis of 4 AGO-OVAR phase 3 trials including patients with advanced epithelial ovarian cancer who underwent upfront surgical management followed by 6 cycles of platinum-based chemotherapy. Of 145 patients with LGSOC, those who underwent complete cytoreduction had significantly improved OS (HR 0.14) compared to those with suboptimal cytoreduction [16]. Expert consensus favors attempting cytoreduction even if the probability of complete gross resection is low [3••, 7]. Neoadjuvant platinum-based chemotherapy is not the favored approach for LGSOC given its relative chemoresistance and association with worse PFS [16, 17, 24–26]. However, a recently presented abstract from a phase 2 pilot study of neoadjuvant fulvestrant plus abemaciclib in patients with LGSOC demonstrated 47% ORR, indicating that neoadjuvant hormonal/targeted therapy combinations may be beneficial for patients when primary cytoreduction is not feasible [27•].

Secondary cytoreduction at time of recurrence should be strongly considered, especially if complete gross resection is achievable. In a retrospective review of 41 recurrent LGSOC patients by Crane et al., median PFS following secondary cytoreduction was significantly improved for patients with no gross residual disease compared to those with residual disease (60.3 vs 10.7 months; p = 0.008), and OS was also significantly improved (93.6 vs 45.8 months; p = 0.04) [28]. A meta-analysis of patients with LGSOC reported that survival was superior for patients whose initial treatment at time of recurrence was surgery as compared to chemotherapy [29]. However, patient selection for secondary cytoreduction in the setting of recurrent LGSOC is not well defined, as most patient selection criteria are based on studies of patients with HGSOC. In general, the gynecologic oncologist is encouraged to consider the disease-free interval and number of recurrence sites when determining eligibility for secondary cytoreduction [30]. Expert consensus recommends a lower threshold for surgical management of recurrent LGSOC given the indolent nature of disease and likely survival benefit even if complete gross resection is not achieved [3••].

### Chemotherapy

Patients with LGSOC were included in historical Gynecologic Oncology Group (GOG) trials that defined platinum-based chemotherapy as the standard of care for ovarian cancer patients following surgical cytoreduction [31–33]. As a result, carboplatin and paclitaxel have become the recommended cytotoxic regimen in the upfront setting. Recommended dosing of carboplatin is an area under the curve of 5–6, and paclitaxel 175 mg/m2 on day 1 of every 21 day cycle, as administered in the control arm of GOG 218 [32]. Dose reductions or discontinuation of therapy may occur for performance status and/or treatment-related toxicities. Myelosuppression is the dose-limiting toxicity associated with carboplatin, with up to 20% of patients experiencing grade 3–4 thrombocytopenia [32–35]. Neurotoxicity is another dose-limiting toxicity associated with paclitaxel, where peripheral neuropathy may be permanent [36].

Based upon clinical practice and ancillary analyses of early ovarian cancer trials, it has become more apparent that those patients with HGSOC drive the response seen to platinum-based chemotherapy. An exploratory case–control study of the AGO-meta-database looked at 4 randomized phase 3 clinical trials where ovarian cancer patients were treated with first-line platinum-based chemotherapy. Of 145 patients with LGSOC, 39 had a suboptimal debulking and response that was able to be evaluated. An overall response rate (ORR) of only 23% was noted, compared to an ORR of 90% in a control cohort of high-grade serous ovarian cancer (p < 0.001) [16]. Other studies have reported even lower response rates (ORR 4%) of LGSOC to cytotoxic chemotherapy [15, 17].

Other non-platinum-based cytotoxic chemotherapies have also been studied in LGSOC prospectively as the standard of care control arms in GOG 281 and MILO/ENGOT-ov11. Physicians choice chemotherapy included either weekly paclitaxel (80 mg/m2 IV on days 1, 8, 15 of every 28 day cycle), liposomal doxorubicin (40–50 mg/m2 IV on day 1 of every 28 day cycle), or topotecan (either 4 mg/m2 on days 1, 8, 15 of every 28 day cycle, or 1.25 mg/m2 on days 1–5 of every 21 day cycle). Response rates to each of these regimens were low, ranging from 0–15% [37••, 38•]. The most common toxicities related to these regimens included abdominal pain (17%), nausea/vomiting (11%), anemia (10%), and palmar plantar erythrodysesthesia (5%). Given the toxicity risk associated with cytotoxic chemotherapy and lack of substantial response in the setting of LGSOC, alternative treatments have emerged including anti-angiogenesis, hormonal therapy, and targeted therapies.

### Anti-angiogenics

Bevacizumab is a monoclonal antibody that binds and prevents the activity of vascular endothelial growth factor (VEGF). Its use in LGSOC may be considered in the primary setting in combination with platinum-based chemotherapy and continued as maintenance, or in the recurrent setting either as combination or monotherapy treatment.

Several large clinical trials have demonstrated a progression-free survival (PFS) benefit with the addition of bevacizumab for frontline treatment of advanced-stage ovarian cancer, although again most patients included in these trials had HGSOC. In the phase III trial GOG 218, Burger et al. demonstrated the addition of bevacizumab to platinum-based chemotherapy, followed by maintenance bevacizumab, provided a 4-month PFS benefit [32]. Oza et al. reported a 2-month PFS improvement when bevacizumab was added to platinum-based chemotherapy in the frontline setting in a phase III randomized trial, ICON 7 (median PFS 24.1 vs 22.4 months for chemotherapy alone, p = 0.04). Only 80 patients in this trial had LGSOC and in this small subgroup, there was not a demonstrated survival benefit [39]. Additionally, the OCTAVIA trial was a single-arm, open-label, international study that demonstrated 12-month PFS 85.6% with the addition of bevacizumab to platinum-based chemotherapy and continued as maintenance for patients with newly diagnosed ovarian cancer. Only 22 (12%) of the study population had LGSOC or endometrioid histology, and these were not separately analyzed [40].

In the recurrent setting, the addition of bevacizumab to chemotherapy has demonstrated improvement in PFS when compared to chemotherapy alone in large phase III clinical trials [41, 42]. In one of these trials (OCEANS), histologic grade was not reported, and in the other (AURELIA), low-grade histology only comprised 5% of the patient population and was not separately evaluated. Retrospective studies have looked at bevacizumab specifically in LGSOC patients. A review of 17 patients with recurrent LGSOC or serous borderline tumors reported an ORR of 55% for those patients with LGSOC who received bevacizumab alone (n = 2) or in combination (n = 15) [43]. Rose et al. reported a low ORR (8.3%) in 12 LGSOC patients treated with bevacizumab, however clinical benefit was significant with most patients achieving long duration of stable disease, and median PFS was 48 months [44]. Dalton et al. reviewed 40 patients with LGSOC undergoing 45 different treatment regimens with the addition of bevacizumab and found an ORR of 47.5%, median PFS 10.2 months, and median overall survival (OS) 34.6 months [45]. Based on these reports, expert consensus and guidelines recommend consideration of the addition of bevacizumab in the treatment of LGSOC [3••, 6, 7].

Bevacizumab is administered intravenously at 10 mg per kilogram (mg/kg) of body weight every two weeks or 7.5–15 mg/kg every three weeks. Contraindications include the presence of a fistula, patients at high risk of bleeding, or those within four weeks of major surgery. Common side effects include hypertension, proteinuria, arthralgias, rhinorrhea, and epistaxis. Less common, but significant side effects include gastrointestinal perforation, fistula, bleeding, and venous thromboembolism [40–42, 46]. Bevacizumab costs $184 to $239 per milliliter of medication [47]. Bevacizumab can interact with various medications. Notably, it enhances the effects of myelosuppressive agents, such as cytotoxic chemotherapy. Additionally, it can worsen the cardiotoxic effect of anthracyclines and should not be administered in combination [47].

### Hormonal Therapy

Hormonal therapy may be utilized in the primary treatment, primary maintenance, and recurrent treatment settings for patients with LGSOC.

Retrospective data supports the use of hormonal therapy as a maintenance strategy for LGSOC. In a large retrospective review of 203 patients with surgical stage II-IV LGSOC, Gershenson et al. reported outcomes of patients who underwent surveillance (n = 133) versus hormonal maintenance (n = 70) following adjuvant platinum-based chemotherapy. Hormonal therapy included a variety of medication regimens: 57% aromatase inhibitor (54% letrozole, 3% anastrazole), 29% tamoxifen, 7% leuprolide, 3% leuprolide and letrozole, 3% leuprolide and tamoxifen, and 1% depo-provera. Median PFS was 64.9 months in those who received hormonal therapy and 26.4 months in those who had undergone surveillance, an almost 40-month difference (p < 0.001). Notably, there was no difference in OS (102.7 vs. 115.7 months) [48]. In another retrospective study by Fader et al., 27 patients with stage II-IV LGSOC received hormonal monotherapy after cytoreductive surgery. Most tumors (96%) had positive ER expression on immunohistochemistry, and 32% had positive PR expression. Hormonal treatment options included letrozole (55%), anastrozole (37%), and tamoxifen (7.4%). Over a median follow-up of 41 months, only 22% of patients recurred, and 3-year PFS and OS were 79% and 93%, respectively [49]. Based on results of these two retrospective studies, a phase III trial, NRG-GY019 (NCT04095364) was developed to evaluate the noninferiority of hormonal monotherapy (letrozole) compared to standard platinum-based chemotherapy followed by letrozole maintenance in the frontline treatment of patients with stage II-IV LGSOC. This trial is currently ongoing and has the potential to change the standard of care for LGSOC treatment in the upfront setting.

The use of hormonal therapy in the recurrent setting is supported by clinical benefit demonstrated in one prospective trial and several retrospective studies. The PARAGON study is a prospective, single-arm phase II trial that evaluated the aromatase inhibitor anastrazole in the setting of recurrent/metastatic ER/PR positive LGSOC and serous borderline ovarian tumors. Most patients (34 of 36) enrolled had LGSOC. Treatment was anastrazole 1 mg daily. While ORR was relatively low at 14%, clinical benefit was observed in 61% of patients, with median duration of benefit 9.5 months, and the regimen was well-tolerated [50]. Additionally, in a single-institution retrospective review by Gershenson et al., 64 patients with LGSOC were reported to have received 89 different regimens of hormonal therapy (aromatase inhibitor, selective estrogen receptor modulator, GnRH agonist, progestin, or a combination of these). ORR was again low at 9%, however 62% had stable disease resulting in a clinical benefit rate of 71%. This was most pronounced for those patients with platinum-sensitive recurrence (disease recurrence 6 months or more following last platinum-based chemotherapy), with clinical benefit rate of 83% as compared to 54% for those with platinum-resistant disease. Additionally, time to progression was longer in patients with ER/PR positive tumors, indicating this may be a predictive marker for treatment [19]. As this has not been further validated in prospective studies, clinical consensus recommends against using ER/PR status as a driving factor in decision to treat LGSOC with hormonal therapy [3••].

The optimal hormonal therapy regimen remains unknown for both primary and recurrent disease. However, in GOG 281, patients in the standard of care treatment arm were eligible to receive hormonal therapy in the form of letrozole (2.5 mg daily) or tamoxifen (20 mg twice daily). Those who received letrozole demonstrated a superior response rate of 14% as compared to 0% with tamoxifen use [37••]. Common hormonal therapy regimens utilized in the treatment of LGSOC including class, dosing, cost, common side effects, and medication interactions are included in Table 1.

**Table 1:** Commonly used hormonal and targeted therapies for recurrent low grade serous carcinoma of the ovary

Medication	Medication Class	Route, Dose, Frequency	Cost ($) per tablet/per month**	Side effects	Medication Interactions
Letrozole	AI	PO 2.5 mg daily	Per tablet (2.5 mg): 10-31 Per month: 291-853	Decreased bone density, arthralgias, insomnia, nausea, edema, headaches, elevated cholesterol, hot flushes	*Can interact with CYP2A6-mediated metabolism
Anastrozole	AI	PO 1 mg daily	Per tablet (1 mg): 7-65 Per month: 194-1,820	Decreased bone density, arthralgias, insomnia, nausea, edema, headaches, elevated cholesterol, hot flushes	Estrogens, estrogen derivatives, and tamoxifen can diminish the therapeutic effect of anastrazole
Tamoxifen	SERM	PO 20 mg BID	Per tablet (20 mg): 4 Per month: 224	Nausea, gastrointestinal disturbances (diarrhea or constipation), insomnia, anxiety, bone and muscle pain, dizziness, hot flashes	*Can interact with CYP-mediated metabolism: -i.e. can increase serum concentration of Vitamin K antagonists (Wafarin) -should not be used concomitantly with anastrozole
Trametinib	MEK Inhibitor	PO 2 mg daily	Per tablet (2 mg): 542 Per month: 15,176	Acneiform rash, GI disturbances (diarrhea, nausea, emesis), hypertension, fatigue, anemia Rare: Decreased cardiac EF, pneumonitis, visual disturbances (I.e. blurry vision, vision loss, color spots)	Enhances the hyponatremic effect of desmopressin and associated agents (i.e. amiloride, acetazolamide); capable of elevating serum concentration of dabrafenib
Selumetinib	MEK Inhibitor	PO 50 mg BID	Per tablet (25 mg): 270 Per month: 30,240	Acneiform rash, diarrhea, fatigue, myalgias Rare: Decreased cardiac EF, visual disturbances (blurry vision, vision loss, color spots)	Can interact with CYP3A4 metabolism and concentration can be altered by other CYP3A4 mediators (i.e. rifampin)
Binimetinib	MEK Inhibitor	PO 45 mg BID	Per tablet (15 mg): 98 Per month: 16,464	Acneiform rash, GI disturbances (diarrhea, nausea, emesis), elevated blood CPK, fatigue, peripheral edema Rare: Decreased cardiac EF, palmar-plantar erythrodysesthesia syndrome, alopecia, stomatitis, dry skin	Serum concentration can be altered by Mitapivat+

Abbreviations: AI, Aromatase Inhibitor; PO, oral; Mg, milligrams; BID, two times a day; SERM, selective estrogen receptor modulator; CPK, creatinine phosphokinase; EF, ejection fraction

*Interaction with CYP-mediated metabolism can lead to an increase or decrease in other medications or an increase or decrease in the level of the medication itself. **month cost calculated by 28 days. +has only been demonstrated in vitro

*References for table: Dowsett et al. *[51]*, Dowsett et al. *[52]*, Soltamox: prescribing information *[53]*, Arimidex: prescribing information *[54]*, Binkhorst et al. *[55]*, Gershenson et al. *[37••]*, Monk et al. *[38•]*, Farley et al. *[56]*, Lexicomp Drug Interactions: Trametinib, Selumetinib, Binimetinib; Dymond et al. *[57]

### MEK Inhibitors

LGSOC tumors are characterized by frequent *MAPK* pathway alterations, including mutations of *KRAS* (27%), *BRAF* (13%), and *NRAS* (9%) [18, 58–62]. This has prompted investigation of mitogen-activated extracellular signal-regulated kinase (MEK) inhibitors, which target a downstream protein kinase of the *MAPK* pathway, as a potential therapeutic target. Several recent clinical trials have demonstrated improved survival of patients with recurrent LGSOC treated with a MEK inhibitor when compared to standard of care chemotherapy or hormonal therapy. Based on sub-analyses of these studies, whether the presence of a *MAPK* pathway alteration improves response to MEK inhibitor remains unclear, supporting MEK inhibitor use regardless of mutational status [3••, 37••, 38•, 56, 63•].

In GOG 281/LOGS, a randomized, multicenter, phase II/III clinical trial, Gershenson et al. compared the MEK inhibitor trametinib to standard-of-care chemotherapy or hormonal therapy in patients with recurrent LGSOC. In the standard of care arm, physician’s treatment choice included either paclitaxel, doxorubicin, topotecan, letrozole, or tamoxifen. Those who were randomized to trametinib received 2 mg orally daily until unacceptable toxicity or disease progression. Patients enrolled (n = 260) were divided evenly among treatment arms. Over a median follow-up of 31 months, trametinib was associated with a significant improvement in PFS when compared to standard of care treatment (median PFS 13 vs 7.2 months; HR 0.48; p < 0.001). The benefit of trametinib remained when compared against each treatment type in the standard of care arm separately. Trametinib was associated with an ORR of 26%, and clinical benefit rate of 85%. Notably, only 22% of patients had a KRAS, BRAF, or NRAS mutation in each arm. While patients with a *RAS* or *RAF* mutation compared to wild type had a greater ORR to trametinib (ORR 50% versus 8%), this was not statistically significant (p = 0.11), and the study was not powered to detect this difference. Additionally, mutation status was not found to be predictive for PFS (p for interaction 0.72). Together, results of this trial support the benefit of trametinib regardless of *MAPK* pathway mutational status. Trametinib was relatively well-tolerated when compared to standard of care therapy. The most common grade 3 to 4 adverse events in the trametinib group were rash (13%), anemia (13%), hypertension (12%), diarrhea (10%), nausea (9%), and fatigue (8%) [37••].

Two other clinical trials have evaluated MEK inhibitors in recurrent LGSOC. GOG 239 was a phase II single-arm trial that evaluated selumetinib in patients with recurrent LGSOC with measurable disease. Fifty-two patients received 100 mg twice daily until disease progression or unacceptable toxicity. Selumetinib demonstrated activity with ORR 15.4% and clinical benefit rate 81%. While the treatment regimen was reported to be well-tolerated, 25% of patients discontinued study treatment due to toxicity and grade 3–4 adverse events included cardiac (1), pain (1), pulmonary (1), gastrointestinal (13), dermatologic (9), and metabolic (7). Tumor tissue was available for testing for 34 patients, and *BRAF* (6%), *KRAS* (41%), other *RAS* (21%) mutations were observed. Mutational status was not associated with a significant difference in ORR [56]. MILO/ENGOT-ov11 was a phase III trial where patients with persistent/recurrent LGSOC with measurable disease were randomized 2:1 to binimetinib 45 mg twice daily or physician’s choice chemotherapy (paclitaxel, topotecan, or pegylated liposomal doxorubicin). Three-hundred and three patients were enrolled. At the time of interim analysis, median PFS did not significantly differ between treatment arms (9.1 months binimetinib versus 10.6 months control), resulting in early study closure according to the prespecified futility boundary. Tumor testing was available for analysis for 215 patients, of which 33% had a *KRAS* mutation, distributed evenly between treatment arms. Interestingly, the presence of a *KRAS* mutation was associated with improved median PFS when patients were treated with binimetinib (17.7 months vs 10.8 months, p = 0.006), as well as improved ORR (OR 3.4, 95% CI 1.53–7.66), indicating that *KRAS* mutation may be predictive of response to binimetinib. *KRAS* mutation in the control arm was not associated with a significant difference in survival [38•]. MEK inhibitor regimens utilized in the treatment of LGSOC including class, dosing, cost, common side effects, and medication interactions are included in Table 1.

Additional research regarding MEK inhibitors in LGSOC is ongoing. RAMP 201 is a phase II randomized trial evaluating the efficacy and safety profile of the MEK inhibitor avutometinib alone or in combination with defactinib, a small molecule inhibitor of focal adhesion kinase (FAK) in patients with recurrent LGSOC. Preliminary data demonstrated a high disease control rate (90% for 8 weeks or longer) for both monotherapy and combination therapy, with both arms having a tolerable safety profile [63•].

### Cyclin-dependent Kinase (CDK) 4/6 and PI3KCA Inhibitors

In addition to *MAPK* pathway alterations, there is data to support involvement of cyclin-dependent kinase (*CDK*) inhibitor deletion and *PIK3CA* alterations in the pathogenesis of LGSOC, highlighting another potential therapeutic target [10, 64]. Recent data presented from a pilot phase II trial demonstrated activity of the *CDK* 4/6 inhibitor abemaciclib in combination with fulvestrant in the neoadjuvant setting for patients with unresectable stage III-IV LGSOC. Patients subsequently underwent interval cytoreductive surgery and received four additional cycles of abemaciclib and fulvestrant, followed by letrozole as maintenance therapy. Cobb et al. enrolled a total of 15 patients: 7 (47%) had a partial response and 5 (33%) had stable disease. Although only a pilot study, this treatment regimen is promising especially in the setting of advanced-stage unresectable disease, in particular given known poor response rate of LGSOC to standard platinum-based neoadjuvant chemotherapy [24, 25, 27•].

Ongoing trials evaluating *CDK* inhibitors in recurrent LGSOC include GOG 3026 and PARAGON II. GOG 3026 is a phase II trial investigating the *CDK* 4/6 inhibitor ribociclib in combination with letrozole. Preliminary results reported in March 2023 demonstrated activity of the regimen, with an ORR of 23% and clinical benefit rate of 79%. Overall, the combination was well-tolerated with the most common grade 3 events being neutropenia (44%) and leukopenia (8%) [65]. PARAGON II is a phase II, non-randomized, open-label study that is currently enrolling patients with advanced/recurrent hormone receptor positive gynecologic malignancy. Treatment arms include letrozole with either a CDK4/6 inhibitor (ribociclib) or a *PIK3CA* inhibitor (alpelisib), and patients are allocated based on *PIK3CA* mutational status [66].

In the above phase II trial conducted by Cobb et al., abemaciclib was administered at a dose of 150 mg orally twice daily, a dose that has been established as efficacious in hormone-receptor-positive breast cancer [27•, 67]. In both GOG 3026 and PARAGON II, ribociclib is given at 600 mg daily on days 1–21 of a 28 day cycle [68]. Alpelisib is given as an oral tablet 300 mg daily continuously. Side effects of *CDK* 4/6 inhibitors include but are not limited to fatigue, myelosuppression, nausea, gastrointestinal disturbances, and alopecia. In a large study of hormone-receptor-positive breast cancer with over 3,000 patients, grade three to four adverse events included neutropenia, increased liver transaminases, and QT prolongation [68]. *CDK* 4/6 inhibitors are a substrate of CYP3A4-mediated metabolism and therefore can interact with all drugs metabolized through this pathway [69, 70]. For abemaciclib, the cost of one 150 mg tablet is $311.44 and for ribociclib the cost is $311.91 to $374.29 for one 200 mg tablet. For alpelisib, the cost of one 150 mg tablet is $437.29. This would amount to $8,720.32 to $47,160.54 for one month of a CDK4/6 inhibitor without consideration of insurance coverage [71].

### Emerging Therapies

There are several ongoing phase II/III clinical trials that are studying the efficacy of hormonal therapy, immunotherapy, *CDK4/6* inhibitors, MEK inhibitors, and other targeted therapies for patients with LGSOC, both in the first-line and recurrent settings (Table 2). Results of these trials are highly anticipated and have the potential to change the standard of care for LGSOC treatment by replacing cytotoxic chemotherapy with more targeted therapeutic options, particularly in the frontline setting.

**Table 2:** Ongoing phase II/III clinical trials in low grade serous carcinoma of the ovary

Trial Name and Identifier	Phase	Treatment Setting	Drug Class	Treatment Arms	Status
NRG-GY019 (NCT04095364)	III	Adjuvant	Hormonal therapy	Control: carboplatin/paclitaxel followed by letrozole maintenance Experimental: letrozole alone	Recruiting
LEPRE (NCT05601700)	III	Adjuvant	Hormonal therapy	Control: carboplatin/paclitaxel Experimental: letrozole alone	Recruiting
ENGOT-ov54/ MATAO (NCT04111978)	III	Adjuvant	Hormonal therapy	Control: placebo maintenance after platinum-based chemotherapy Experimental: letrozole maintenance after platinum-based chemotherapy	Recruiting
NCT03531645	II	Neoadjuvant	CDK 4/6 inhibitor; hormonal therapy	Experimental: abemaciclib + fulvestrant	Active, not recruiting
FUCHSia (NCT03926936)	II	Recurrent	Hormonal therapy	Experimental: fulvestrant	Recruiting
PARAGON II (ACTRN12621000639820)	II	Recurrent	Hormonal therapy; CDK 4/6 inhibitor; PIK3CA inhibitor	Experimental: letrozole + ribociclib Experimental: letrozole + alpelisib	Recruiting
GOG 3026 (NCT03673124)	II	Recurrent	CDK 4/6 inhibitor; hormonal therapy	Experimental: ribociclib + letrozole	Active, not recruiting
ALEPRO (NCT05872204)	II	Recurrent	CDK 4/6 inhibitor; hormonal therapy	Experimental: abemaciclib + letrozole	Not yet recruiting
NCT05113368	II	Recurrent	Multi-kinase inhibitor; hormonal therapy	Experimental: regorafenib + fulvestrant	Recruiting
GOG-3052/ ENGOT-ov60/ RAMP 201 (NCT04625270)	II	Recurrent	MEK inhibitor; FAK inhibitor	Experimental: avutometinib +/- defactinib	Recruiting
RAMP 301 (NCT06072781)	III	Recurrent	MEK inhibitor; FAK inhibitor	Control: investigator’s choice chemo or hormonal therapy Experimental: avutometinib + defactinib	Not yet recruiting
PERCEPTION (NCT04575961)	II	Recurrent	Immunotherapy	Experimental: platinum-based chemotherapy + pembrolizumab	Recruiting
ComboMATCH (NCT05554367)	II	Recurrent	MEK inhibitor; CDK 4/6 inhibitor	Experimental: binimetinib +/- palbociclib	Not yet recruiting
WO42178/ GOG-3051/ ENGOT-GYN2/ BOUQUET (NCT04931342) [72]	II	Recurrent	Targeted therapy	Experimental: multiple biomarker driven arms	Recruiting

## Data Availability

No datasets were generated or analysed during the current study.
